# Catching common carp with eDNA in Thailand’s rivers

**DOI:** 10.1016/j.isci.2025.114583

**Published:** 2025-12-31

**Authors:** Maslin Osathanunkul, Sarawut Ounjai, Rossarin Osathanunkul, Panagiotis Madesis

**Affiliations:** 1 Department of Biology, Faculty of Science, Chiang Mai University, Chiang Mai, Thailand; 2 Faculty of Economics, Chiang Mai University, Chiang Mai, Thailand; 3 Institute of Applied Biosciences, Centre for Research & Technology Hellas (CERTH), Thessaloniki, Greece; 4 Laboratory of Molecular Biology of Plants, Department of Agriculture, Crop Production and Rural Environment, University of Thessaly, Volos, Magnesia, Greece

**Keywords:** Ecology, Environmental biotechnology, Ichthyology, Aquatic biology

## Abstract

Common carp (*Cyprinus carpio*) are both ecologically disruptive invaders and economically important aquaculture species in Thailand. This study applied environmental DNA (eDNA) analysis with digital PCR (dPCR) and Bayesian occupancy modeling to assess carp distribution across five major river systems from 2022 to 2024. eDNA was detected at 80% of surveyed sites in 2022, increasing to 96% by 2024, with higher concentrations particularly in the Nan and Ping rivers. Occupancy probabilities remained relatively stable across years, while rising eDNA concentrations indicate greater detectability and possible local increases in biomass. Detection probabilities were high at both field and laboratory stages, confirming methodological reliability. While aquaculture escapes and hydrological drivers are plausible influences, these factors were not directly tested. The findings provide robust baseline evidence of widespread persistence of common carp in Thailand’s rivers and highlight the potential of eDNA as a scalable, cost-effective tool for freshwater monitoring and management.

## Highlights

• eDNA reveals widespread common carp across Thailand’s rivers
• Detection rates increased from 2022 to 2024 while occupancy remained stable
• False-positive-aware occupancy modeling improves confidence in detections
• eDNA offers scalable monitoring for invasive fish in tropical river systems

## Graphical abstract

**Figure:**
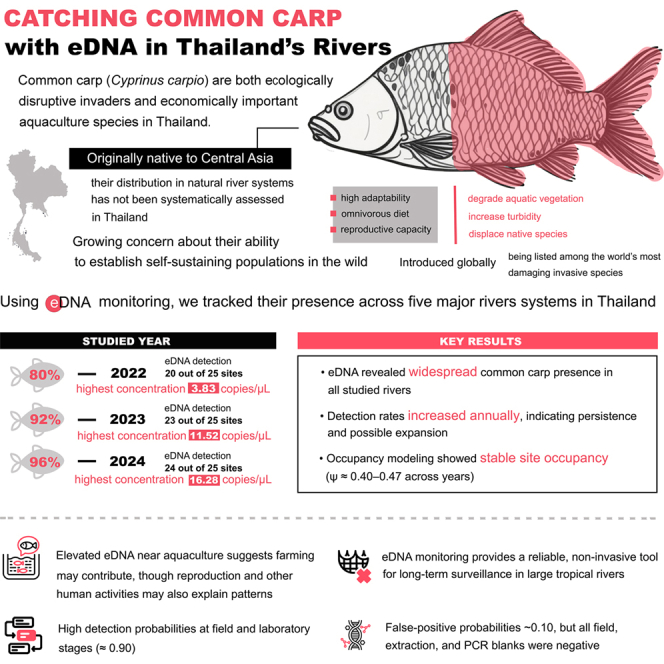

## Introduction

Invasive species are a leading driver of biodiversity loss in freshwater ecosystems, disrupting food webs, degrading habitats, and outcompeting native species. These impacts often result in long-term ecological and economic damage.^1^^,^^2^ Early detection is critical for effective management, but conventional survey methods such as visual observation, netting, and electrofishing are often labor-intensive, costly, and ineffective for detecting low-density or elusive species.^3^

Environmental DNA (eDNA) has emerged as a sensitive and non-invasive tool for aquatic biodiversity monitoring. By detecting trace DNA shed by organisms into the environment, eDNA enables species detection even when individuals are rare or cryptic.^4^ Its scalability and adaptability allow for application in diverse ecosystems, including alpine streams, tropical rivers, and marine environments.^5^^,^^6^ eDNA methods are increasingly used to support conservation decision-making and invasive species control efforts.^7^^,^^8^

The common carp (*Cyprinus carpio*) exemplifies the complex challenge of managing invasive species with economic importance. Originally native to central Asia and among the earliest domesticated fish,^9^ carp have been introduced worldwide for aquaculture, ornamental use, and recreational fishing. Despite being listed among the world’s most damaging invasive species due to their disruptive feeding and habitat-altering behavior,^10^ they are also economically and culturally significant in many regions, including Thailand.^11^

In Thailand, common carp are actively promoted for aquaculture by the Department of Fisheries. However, their distribution in natural river systems has not been systematically assessed. Given their high adaptability, omnivorous diet, and reproductive capacity, there is growing concern about their ability to establish self-sustaining populations in the wild. Carp are known to degrade aquatic vegetation, increase turbidity, and displace native species.^12^^,^^13^

eDNA monitoring provides an efficient approach to track carp distribution in large and hydrologically complex rivers. Species-specific assays have enabled effective carp surveillance in North America, Europe, and Australia using eDNA tools.^14^^,^^15^^,^^16^ Recent developments also allow eDNA data to be combined with biodiversity indices and spatial models for improved ecological interpretation.^17^^,^^18^

This study investigates the distribution of common carp in five major river systems in Thailand: the Ping, Wang, Yom, Nan, and Chao Phraya rivers. Over a three-year period from 2022 to 2024, we applied eDNA-based methods to assess temporal and spatial trends. The findings provide essential baseline data for future freshwater management and the sustainable integration of aquaculture and biodiversity conservation.

## Results

This study reports findings from a three-year eDNA survey targeting common carp across five major river systems in Thailand. The results reveal spatial and temporal patterns in species presence, as inferred from both eDNA concentrations and site occupancy probabilities. By integrating field-level detection, laboratory amplification, and probabilistic modeling, we evaluated detection trends, occupancy estimates, and methodological performance over time.

### Common carp detected in the majority of sites across all river systems

**Figure 1:**
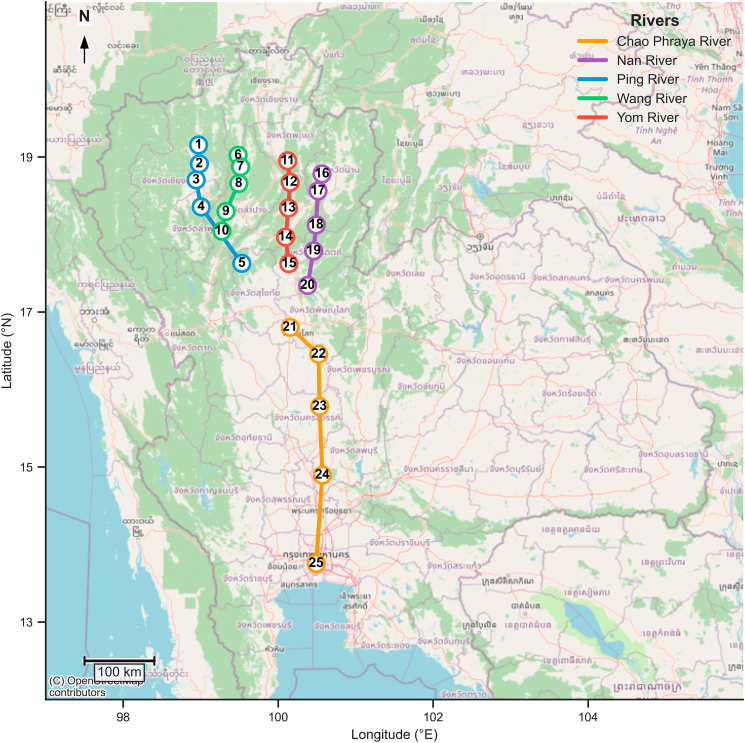
Map showing sampling sites in five major rivers (Ping, Wang, Yom, Nan, and Chao Phraya) in Thailand

Over the three-year survey period from 2022 to 2024, eDNA analysis revealed widespread presence of common carp in all five studied rivers: the Ping, Wang, Yom, Nan, and Chao Phraya Figure 1. At each site, triplicate water samples were collected annually, totaling 75 samples per year and 225 samples across the full study.

**Figure 2:**
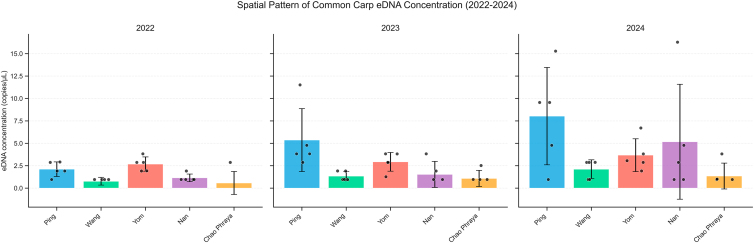
An average eDNA detection over the study period (2022–2024) of each studied river system

Detection rates increased over time. In 2022, common carp eDNA was detected at 20 out of 25 sites (80%). By 2023, detections occurred at 23 sites (92%), and in 2024, 24 sites (96%) yielded positive results. Only site 25, located in the lower Chao Phraya river, remained consistently negative across all three years despite its proximity to positive detections at sites 21–24. The Nan river recorded the highest eDNA concentration in 2024 at 16.28 copies per microliter, followed closely by the Ping river with a peak of 15.29 copies per microliter. In contrast, the lowest detection frequencies and concentrations were generally found in the Chao Phraya and Wang rivers. A summary of eDNA concentrations by site and year is provided in Table 1, while Figure 2 illustrates river-level averages.

**Table 1:** eDNA concentration of common carp (*Cyprinus carpio*) detected across sampling sites in major rivers (Ping, Wang, Yom, Nan, and Chao Phraya) of Thailand during a three-year monitoring period (2022–2024)

Site	River	eDNA concentration (copies/uL)		
		2022	2023	2024
1	Ping	2.867	3.825	9.561
2		1.913	2.871	4.781
3		2.915	3.830	9.558
4		1.912	11.520	15.290
5		0.956	4.781	0.959
6	Wang	0.959	1.919	2.869
7		0.956	0.957	0.957
8		0.955	1.912	2.881
9		0.959	0.956	2.869
10		–	0.983	0.957
11	Yom	1.935	1.274	1.918
12		1.913	2.872	3.013
13		2.868	3.824	2.867
14		3.830	2.872	6.708
15		2.870	3.828	3.823
16	Nan	0.961	0.957	0.960
17		0.957	3.826	2.866
18		0.956	0.956	0.956
19		1.912	–	4.780
20		0.956	1.912	16.280
21	Chao Phraya	–	0.958	0.956
22		2.868	2.546	3.822
23		–	0.965	0.957
24		–	0.955	1.008
25		–	–	–

### Annual increases in eDNA concentrations suggest local population growth

Analysis of temporal patterns revealed notable increases in eDNA concentrations over time, particularly in the Ping and Nan rivers. In the Ping river (sites 1–5), sites 1 through 4 showed consistent increases across all three years, while site 5 peaked in 2023 before substantially declining in 2024. The Wang river (sites 6–10) exhibited moderate increases at sites 6, 8, 9, and 10, whereas site 7 remained stable with low-level detections.

**Figure 3:**
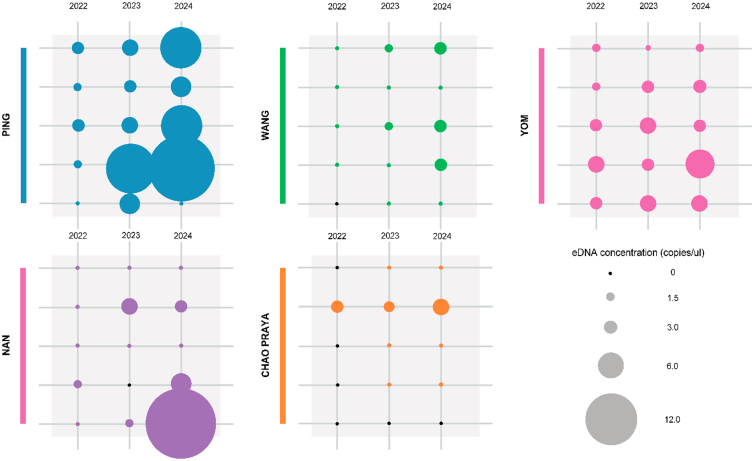
eDNA concentration of each studied river system compared between the studied years (2022–2024). Point sizes are proportional to mean eDNA concentration. Colors indicate river systems: blue (Ping), green (Wang), pink (Yom), purple (Nan), orange (Chao Phraya)

In the Yom river (sites 11–15), site 12 exhibited gradual annual increases, site 13 peaked in 2023 before declining in 2024, and site 14 showed a rebound after a dip in 2023. Sites 11 and 15 remained relatively stable. The Nan river (sites 16–20) displayed a sharp and continuous increase at site 20, along with rising concentrations at sites 17 and 19. Sites 16 and 18 yielded low but consistent levels across all years. The Chao Phraya river (sites 21–25) showed little change, with sites 21–24 displaying consistently low concentrations, and site 25 remaining negative throughout (Figure 3).

These findings suggest that in several locations, especially in the Ping and Nan river systems, common carp populations may be expanding or becoming more detectable due to either increased biomass or improved environmental conditions for eDNA persistence.

### Statistical validation of spatial and temporal patterns

Statistical validation confirmed significant spatial and temporal differences in carp eDNA concentrations. A two-way ANOVA on log-transformed concentrations revealed main effects of river (F[4,60] = 11.91, *p* = 3.5 × 10^−7^, partial η^2^ = 0.44) and Year (F[2,60] = 7.94, *p* = 8.7 × 10^−4^, partial η^2^ = 0.21), with no river × year interaction (F[8,60] = 0.61, *p* = 0.768) (Table S1). Post-hoc Tukey HSD comparisons showed that the Ping and Yom rivers consistently had higher concentrations than the Chao Phraya and Wang rivers (e.g., Ping > Chao Phraya, *p* < 0.000001; Yom > Wang, *p* = 0.00009), while concentrations in 2024 were significantly greater than in 2022 (*p* = 0.00054) (Table S2). Model assumptions were satisfied (Shapiro-Wilk W = 0.966, *p* ≈ 0.21; Levene’s test for river, *p* = 0.144; for year, *p* = 0.621) (Table S3). Robustness checks using ART-ANOVA, Kruskal-Wallis, Friedman, and mixed-effects models produced concordant results (Table S4). Global Moran’s I further indicated significant spatial autocorrelation of site-level mean concentrations (I = 0.227, z = 2.20, *p* = 0.0137; permutation *p* = 0.022), confirming that eDNA signals were spatially clustered rather than randomly distributed (Table S5).

### Site occupancy remained stable across years despite variable concentrations

Bayesian occupancy modeling was applied to estimate the probability of site occupancy (ψ) and to distinguish true species presence from potential detection error. Site occupancy probabilities were moderately high across all years, with posterior mean estimates of 0.403 in 2022, 0.470 in 2023, and 0.474 in 2024. Although ψ values varied slightly across years, the differences fall within broad uncertainty bounds and do not indicate biological decline. Instead, they reflect model variability while confirming widespread persistence of common carp across surveyed areas.

Field-level true detection probabilities (θ_11_) were consistently high, ranging from 0.899 to 0.901, and laboratory-level true detection probabilities (p_11_) were similarly stable around 0.900 across all years. False-positive probabilities at both the field (θ_10_) and laboratory (p_10_) stages were ∼0.10, with tight credible intervals (0.095–0.105). Collectively, these values indicate that the eDNA workflow provided robust detection performance (Table 2). The stability of detection probabilities alongside moderate but consistent occupancy estimates highlights the reliability of the model framework.

**Table 2:** Posterior summaries of the probabilities of occurrence and detection, including the probability of false positives and false negatives, at both the field and laboratory stages of the analyses. Values are posterior means with 95% credible intervals (2.5%–97.5%) shown in brackets

Parameter	Posterior mean		
	2022	2023	2024
Occurrence (ψ)	0.403 [0.016, 0.971]	0.470 [0.014, 0.979]	0.474 [0.016, 0.979]

**Probability of true positives**			

Field (θ11)	0.899 [0.895, 0.904]	0.899 [0.895, 0.904]	0.901 [0.897, 0.905]
Lab (p11)	0.900 [0.895, 0.905]	0.900 [0.895, 0.905]	0.900 [0.896, 0.904]

**Probability of false positives**			

Field (θ10)	0.100 [0.095, 0.105]	0.100 [0.095, 0.105]	0.100 [0.096, 0.104]
Lab (p10)	0.100 [0.095, 0.105]	0.100 [0.095, 0.105]	0.100 [0.096, 0.104]

When expressed in environmental units, the estimated limits of detection correspond to low-copy thresholds at the scale of river water volume. Although concentrations are reported here as copies/μL of DNA extract, this conversion provides a transparent framework for interpreting detection sensitivity relative to sampling effort and environmental context.

### Environmental covariates and detection processes

In addition to hydrological variation, our recorded water-chemistry covariates showed measurable but parameter-specific influences in the occupancy framework. Posterior inclusion probabilities (PIPs; Table S6) indicated modest effects on occupancy (ψ) (PIPs ≈0.424–0.664; highest for conductivity and total dissolved solid [TDS] in 2024: 0.664, 0.650), whereas detection processes were more sensitive to environmental conditions. Field true detection (θ_11_) was most associated with pH and temperature in 2023 (0.917, 0.708), with more even contributions across covariates in 2024 (TDS 0.619, CON 0.591, TEM 0.582, pH 0.502). Field false positives (θ_10_) were consistently linked to TDS and conductivity across all years (0.676–0.757 and 0.692–0.725). At the laboratory stage, true detection (p_11_) showed only moderate covariate signals (≤0.64), while temperature strongly influenced lab false positives (p_10_; 0.825–0.981). These results demonstrate that water chemistry primarily influences detectability and error probabilities rather than overall site occupancy, aligning with our observation that ψ remained stable even as eDNA concentrations increased in some rivers.

### Comparative analysis with provincial fisheries records

To contextualize our eDNA results, we compared mean provincial eDNA concentrations with carp aquaculture production data from the Department of Fisheries, Thailand, offset by one year (e.g., eDNA 2022 vs. production 2021). This temporal alignment reflects that our sampling took place in January–February of each year. Several provinces showed clear correspondence between high aquaculture production and elevated eDNA detections. For example, Chiang Mai exhibited a steady increase in both aquaculture output (6,133 → 7,159 tons) and eDNA concentration (2.40 → 9.80 copies/μL) across 2021–2023. Nakhon Sawan, a major production center (>15,000 tons annually), also yielded some of the highest mean eDNA concentrations in 2024. In contrast, provinces with lower production, such as Tak and Phayao, consistently showed low eDNA signals (<3 copies/μL). These comparisons are summarized in Table S7.

### Reliable molecular workflow ensures consistent detection across conditions

The use of a standardized sampling protocol, combined with internal positive controls and inhibition tests, helped ensure consistent data quality across rivers and years. Digital PCR (dPCR), with its high sensitivity and quantification accuracy, enabled detection even in samples with low eDNA concentrations. Raw partition counts (k and *N*) for all dPCR replicates are provided in Table S8 to ensure transparency of quantification. The application of validated species-specific primers and probes further minimized the risk of cross-amplification or false positives.

Replication at both field and laboratory stages, along with strict contamination controls, supported the robustness of results. Negative controls (field blanks, extraction blanks, and PCR blanks) were consistently negative across all runs, confirming that the ∼0.10 false-positive probabilities estimated by the occupancy model represent stochastic processes rather than contamination events. Together, these methodological strengths underpin the confidence in the observed patterns of carp distribution and support the conclusion that eDNA monitoring is effective for long-term surveillance in large, hydrologically dynamic freshwater systems.

## Discussion

This study provides a comprehensive, multi-year molecular assessment of common carp distribution in Thailand’s major river systems using eDNA analysis. The combination of dPCR and Bayesian occupancy modeling enabled the detection of both spatial and temporal patterns, offering robust insights into the ecological status of this ecologically disruptive but economically valuable species.

### Persistent distribution and signs of population growth

The consistent detection of common carp eDNA across more than 90% of sites over three years indicates that the species is well established throughout the Ping, Wang, Yom, Nan, and Chao Phraya rivers. This pattern indicates widespread persistence of common carp across the basin. Temporal increases in eDNA concentrations, particularly in the Nan and Ping rivers, suggest broader distribution and higher detection probabilities. However, our dataset does not allow us to attribute these patterns to specific processes such as aquaculture escapes or natural dispersal, and multiple drivers including population dynamics, hydrological transport, and environmental variation may contribute.

Although eDNA concentrations increased across years, these signals should not be interpreted solely as biomass increases. Rising concentrations may reflect multiple processes, including population growth, gamete release during spawning, hydrological redistribution, or reduced DNA degradation. Without concurrent capture surveys or biomass assessments, eDNA data cannot distinguish among these mechanisms. Our comparison with provincial aquaculture production records provides contextual support for eDNA trends but does not substitute for traditional biomass estimates. Future monitoring efforts would benefit from combining eDNA surveys with fishery-independent sampling to calibrate eDNA concentrations against organismal abundance.

Although raw detection rates increased from 80% in 2022 to 96% in 2024, site occupancy estimates declined slightly (0.496–0.465). This reflects the distinction between occupancy and detection in hierarchical models: ψ represents the probability that sites are truly occupied, while detection rates reflect observed positives. Because most sites were already positive, occupancy estimates approached saturation, and minor fluctuations are best interpreted as model uncertainty rather than evidence of decline. In contrast, rising eDNA concentrations indicate greater detectability or local biomass increases within already occupied sites.

The site occupancy probabilities remained relatively stable across all years, further supporting the conclusion that common carp are firmly established in these river systems. These findings are consistent with other studies that report long-term persistence and expansion of common carp in invaded habitats, where their ecological impacts include sediment disturbance, vegetation loss, and disruption of native fish assemblages.^12^^,^^13^^,^^19^

### Anthropogenic influences and eDNA as a proxy for abundance

Increases in eDNA concentration were most evident in some provinces with high aquaculture production, such as Chiang Mai and Nakhon Sawan, although this pattern was not consistent across all provinces. For example, Tak and Phayao reported moderate production but exhibited low eDNA detections. These mixed results indicate that aquaculture may contribute to eDNA signals in certain areas, consistent with previous findings that escapes from aquaculture facilities can augment wild populations.^20^^,^^21^ However, other processes such as natural dispersal, hydrological connectivity, and environmental conditions are also likely to play important roles. In Thailand, where aquaculture and capture fisheries often overlap spatially, distinguishing between wild and farm-derived populations remains a challenge. Independent of these sources, several studies have shown that eDNA concentration correlates with organismal abundance or biomass, making it a useful proxy for assessing relative population density.^22^^,^^23^^,^^24^ Future work incorporating molecular integrity indices or allometric scaling approaches may improve the reliability of eDNA-based abundance estimation.^25^

Comparing eDNA detections with province-matched fisheries production data provides an ecologically relevant scale for interpreting our results. In several cases, such as Chiang Mai and Nakhon Sawan, the correspondence was strong, with higher aquaculture outputs associated with higher eDNA concentrations. However, the relationship was not universal, with some provinces (e.g., Tak and Phayao) showing low eDNA despite moderate production. This variation likely reflects hydrological connectivity, farm-to-river discharge practices, and local environmental conditions influencing eDNA transport and persistence. These findings suggest that eDNA signals integrate both biological production inputs and hydrological processes, reinforcing the need for future work combining farm-level records, hydrological measurements, and eDNA monitoring to disentangle the contributions of aquaculture escapees from wild populations.

### Hydrological and environmental factors shaping detection patterns

Hydrological and environmental conditions strongly influence the spatial and temporal patterns of eDNA detection, and their effects must be considered when interpreting results from dynamic tropical river systems. The observed spatial variation in eDNA detection likely reflects a combination of biological and environmental factors. Hydrology strongly influences eDNA transport, retention, and degradation: high-flow conditions dilute signals, whereas slower or impounded waters allow eDNA to accumulate and persist.^26^^,^^27^ Seasonal flooding in tropical regions, such as Thailand, can further redistribute eDNA across sites. Because all sampling was conducted during the dry season (January–February), our results represent stable-flow conditions; monsoon-driven hydrological changes are expected to alter transport and persistence, highlighting the need for future wet-season surveys.

Although flow velocity, UV exposure, and microbial activity were not measured, we incorporated basic water chemistry variables (temperature, pH, TDS, conductivity) into occupancy models. PIP analysis indicated that these covariates contributed more strongly to detection and false-positive processes than to occupancy itself (Table S6). Variability in eDNA concentrations was therefore consistent with mechanisms described in the literature.^28^^,^^29^

From a practical monitoring perspective, these findings suggest that physicochemical conditions such as elevated conductivity or total dissolved solids may reduce eDNA detectability even when target organisms are present. In such settings, adaptive sampling strategies may be warranted, including increasing the number of field or PCR replicates, filtering larger water volumes, or using smaller filter pore sizes to improve capture efficiency. Incorporating these adjustments can enhance detection reliability in tropical river systems where environmental conditions are expected to influence eDNA transport, persistence, and amplification.

Notably, site 25 in the Chao Phraya river yielded consistently negative results across all three years despite its proximity to positive detections at sites 21–24. This localized absence may reflect hydrological barriers, such as altered flow regimes or channel structures, that limit fish movement, or environmental degradation that reduces eDNA persistence. Given the high degree of urbanization in this river segment, pollution and reduced habitat quality are also plausible factors. Further work integrating eDNA surveys with hydrological measurements and water quality assessments will be required to clarify the mechanisms underlying this consistent non-detection.

### Methodological reliability and implications for monitoring

The high detection probabilities at both field and laboratory stages affirm the robustness of the eDNA workflow used in this study. The use of digital PCR provided enhanced sensitivity and reduced susceptibility to PCR inhibition. Standardized filtration, DNA extraction, and quality control procedures helped ensure consistent results.

Posterior estimates indicated high probabilities of true detection at both the field (θ_11_ ≈ 0.90) and laboratory (p_11_ ≈ 0.90) stages, while false-positive probabilities were estimated at ∼0.10 for both stages (θ_10_ and p_10_). Although such values fall within the range reported in other eDNA studies using false-positive occupancy models, a ∼10% false-positive probability is not negligible and must be interpreted with caution. To minimize the risk of contamination, multiple levels of negative controls were implemented, including field blanks prepared on each sampling day, extraction blanks processed with every batch, and PCR blanks included on every plate. None of these controls showed amplification, supporting that the model-estimated false positives reflect stochastic error rather than contamination. These results underscore the importance of robust contamination controls and conservative interpretation of eDNA signals in occupancy modeling.

Detections occurring near the limit of detection (LOD) should therefore be interpreted cautiously in applied monitoring contexts. Given the model-estimated false-positive probabilities at both the field (θ10 ≈ 0.10) and laboratory (p10 ≈ 0.10) stages, isolated low-level detections should not be treated as definitive evidence of local establishment. Instead, confirmation should rely on multiple positive replicates within a sampling event, spatial consistency across nearby sites, and temporal repeatability across survey periods. Such conservative decision thresholds are particularly important for invasive species management and early-warning surveillance and are consistent with best-practice recommendations for LOD-aware interpretation of eDNA data.

This study highlights the utility of eDNA for long-term aquatic monitoring, especially in regions where traditional survey methods are logistically challenging or invasive species may be underreported. The approach offers a cost-effective, scalable solution for biodiversity assessments and supports adaptive management in freshwater systems.^4^^,^^30^

### Management implications and future applications

The application of eDNA for tracking invasive species such as common carp has significant implications beyond ecology, extending into economic, social, and management domains. In Thailand, common carp are widely farmed and contribute to rural livelihoods, especially among small- and medium-scale aquaculture enterprises. However, unintentional releases from aquaculture ponds can lead to the establishment of self-sustaining wild populations, potentially threatening native biodiversity.^20^^,^^21^^,^^31^

From an economic standpoint, eDNA represents a cost-effective and scalable tool for aquatic monitoring. Compared to traditional survey methods, eDNA requires less manpower, enables rapid sample processing, and minimizes disturbance to habitats.^4^^,^^7^ These advantages are particularly valuable in low-resource settings, where limited capacity often constrains routine surveillance of freshwater systems. eDNA also enables proactive management by allowing earlier detection of population spread, which can reduce the long-term costs of invasive species control.^32^

Socially, eDNA-based monitoring supports transparency and participation in freshwater governance. In community-managed conservation zones or co-management frameworks, eDNA results can be used by local stakeholders to validate environmental changes, detect unauthorized species introductions, and advocate for policy responses.^33^^,^^34^ This strengthens accountability and builds trust between communities, scientists, and government agencies. Moreover, eDNA can serve as a communication bridge between ecological science and fisheries economics, helping to align biodiversity conservation with food security and rural development goals.^35^

Future research can further improve management applications. The use of lineage-specific eDNA markers will help differentiate wild from farmed carp populations.^36^^,^^37^ Spatial modeling tools, such as the eDITH R package, can enhance interpretation by accounting for flow connectivity and predicting eDNA distribution beyond sampled locations.^38^ Incorporating molecular integrity indices may also improve the reliability of eDNA as a proxy for relative abundance and help diagnose DNA degradation dynamics.^25^

As freshwater ecosystems in Southeast Asia face mounting pressures from aquaculture expansion, land-use change, hydrological alteration, and climate variability, eDNA offers a timely, socially responsive, and practical tool to support biodiversity conservation and sustainable fisheries management. The data generated through this study provide a strong foundation for national monitoring programs and highlights the need for integrative approaches that balance ecological protection with economic development.

### Limitations of the study

This study provides robust, multi-year evidence of the widespread presence and persistence of common carp across Thailand’s major river systems using eDNA and Bayesian occupancy modeling; however, several limitations should be acknowledged when interpreting the results.

First, all sampling was conducted during the dry season (January–February) in each study year. Consequently, the findings represent relatively stable hydrological conditions and do not capture seasonal variability associated with monsoon-driven high flows, which are known to influence eDNA transport, dilution, and degradation. Wet-season sampling may yield different spatial patterns of detectability and should be incorporated in future monitoring to fully resolve seasonal dynamics.

Second, although increases in eDNA concentration over time were observed at several sites, eDNA signals cannot be directly equated with organismal abundance or biomass. Elevated concentrations may reflect multiple processes, including population growth, spawning activity, hydrological redistribution of DNA, or changes in environmental conditions affecting DNA persistence. Without concurrent capture-based surveys or independent biomass estimates, the mechanisms underlying temporal increases in eDNA concentration cannot be conclusively identified.

Third, while Bayesian occupancy modeling explicitly accounted for imperfect detection and false-positive errors at both field and laboratory stages, detections occurring near the LOD should be interpreted cautiously in applied management contexts. Isolated low-level detections may not represent stable local populations and should ideally be corroborated through repeated sampling, spatial consistency across neighboring sites, or complementary monitoring approaches.

Fourth, the study did not directly quantify hydrological variables such as flow velocity, discharge, or water residence time, nor did it incorporate land-use or point-source discharge data from aquaculture facilities. As a result, the relative contributions of natural dispersal versus aquaculture-related inputs to observed eDNA patterns remain unresolved. Future studies integrating hydrological modeling, farm-level data, and lineage-specific genetic markers would improve discrimination between wild and farm-derived carp populations.

Finally, the spatial resolution of sampling was limited to five sites per river system, providing basin-scale coverage but potentially overlooking fine-scale habitat heterogeneity or localized barriers to movement. Denser spatial sampling, combined with network-based modeling approaches, could further refine understanding of carp distribution and connectivity within complex riverine landscapes.

Despite these limitations, the study establishes a strong baseline for long-term molecular surveillance of common carp in tropical river systems and demonstrates the value of integrating eDNA with hierarchical modeling for large-scale freshwater monitoring.

## Resource availability

### Lead contact

Further information and requests for resources should be directed to and will be fulfilled by the lead contact, Maslin Osathanunkul (maslin.cmu@gmail.com).

### Materials availability

This study did not generate new unique reagents or materials. All primers and probes used in this study have been published previously.^39^^,^^40^ Detailed protocols for eDNA sampling, DNA extraction, and molecular analyses are provided in the STAR Methods section.

### Data and code availability

• All data supporting the findings of this study are provided within the article and the supplemental information. Provincial fisheries production data are publicly available from the Department of Fisheries, Thailand (URL provided in the key resources table).
• The Bayesian occupancy modeling code is publicly accessible via the RShiny application described in Diana et al., 2021.
• This study did not generate new datasets or resources beyond those described above.

## Acknowledgments

This research was partially supported by 10.13039/501100002842Chiang Mai University. We acknowledge the Department of Fisheries, Thailand, for provincial fisheries production data used in this study. We also thank the research team members and field assistants whose efforts in water sampling and site logistics were essential to the success of this project.

## Author contributions

Conceptualization, M.O.; methodology, M.O.; software, M.O. and S.O.; validation, M.O. and P.M.; formal analysis, M.O.; investigation, M.O.; resources, M.O. and R.O.; data curation, M.O., S.O., and R.O.; writing – original draft, M.O.; writing – review and editing, M.O., S.O., R.O., and P.M.; visualization, M.O.; supervision, P.M.; funding acquisition, M.O. and R.O.

## Declaration of interests

The authors declare no competing interests.

## Declaration of generative AI and AI-assisted technologies in the writing process

During the preparation of this work the authors used QuillBot in order to improve readability and language. After using this tool/service, the authors reviewed and edited the content as needed and take full responsibility for the content of the publication.

## STAR★Methods

### Key resources table

**Table:** 

REAGENT or RESOURCE	SOURCE	IDENTIFIER
**Biological samples**		

Environmental DNA samples: Ping, Wang, Yom, Nan and Chao Phraya Rivers	This study	N/A

**Critical commercial assays**		

DNeasy Blood and Tissue Kit	Qiagen	Cat# 69504
OneStep PCR Inhibitor Removal Kit	Zymo Research	Cat# D6030

**Oligonucleotides**		

*Cyprinus carpio* ITS1 primers and probe	Minamoto et al.^39^	N/A

**Software and algorithms**		

R	R Foundation for Statistical Computing	v4.2.3
RShiny occupancy modeling application	Diana et al.^41^	https://shinyapps.io
BLASTn	NCBI	https://blast.ncbi.nlm.nih.gov
QGIS	QGIS Development Team	v3.22

**Data resources**		

Provincial fisheries production data (Thailand)	Department of Fisheries, Thailand	https://www.fisheries.go.th

### Experimental model and study participant details

This study did not involve experimental animals, human participants, cell lines, or plant models. All biological material analyzed consisted exclusively of environmental DNA (eDNA) obtained from surface water samples collected from five major river systems in Thailand (Ping, Wang, Yom, Nan, and Chao Phraya Rivers).

The target organism, common carp (*Cyprinus carpio*), was not directly captured, handled, or experimentally manipulated. Consequently, information regarding sex, age, developmental stage, or husbandry conditions is not applicable. Water sampling was conducted under field survey activities that did not require animal ethics approval, and all procedures complied with local regulations governing environmental sampling.

### Method details

#### Water sampling

Surface water samples were collected from five major rivers in Thailand: the Ping, Wang, Yom, Nan, and Chao Phraya Rivers. A total of 25 sites were sampled, with five locations selected from each river (Figure 1). At each site, one liter of surface water was collected and immediately filtered in the field using a BD Luer-Lok syringe and a 0.7 μm glass fiber filter (Whatman GF/F). The number of filters used per site varied according to water turbidity. Three biological replicates were collected at each site. As a field-negative control, 1000 mL of distilled water were filtered at each location using the same procedure as environmental samples.

Sampling was conducted during the dry season in 2022, 2023, and 2024 (January–February) to maintain consistency across years and avoid monsoon-related hydrological variability. Accordingly, results represent dry-season conditions only.

#### DNA extraction and PCR inhibition test

DNA was extracted using the DNeasy Blood and Tissue Kit (Qiagen, Hilden, Germany) with minor modifications following Osathanunkul and Minamoto.^42^ Each 1 L water sample was filtered through one to three 0.7 μm GF/F filters depending on turbidity, and filters from each replicate were pooled prior to extraction. Filters were incubated with proteinase K at 56°C for 3 h, with the proteinase K volume doubled relative to manufacturer recommendations. DNA was eluted in 100 μL of buffer AE.

All extracts were subsequently treated with the OneStep PCR Inhibitor Removal Kit (Zymo Research), maintaining a final elution volume of 100 μL. PCR inhibition was assessed using an internal positive control (IPC) consisting of a synthetic DNA fragment of the jellyfish *Chiropsoides buitendijki*, a marine species absent from freshwater systems.^43^ IPC primers and probe targeting the 16S rRNA gene were added at 1.5 × 10^2^ copies per reaction. Inhibition was defined as a cycle threshold (Cq) shift ≥3 cycles relative to negative controls, following Hartman et al.^44^ No inhibition was detected in any sample.

#### Digital PCR (dPCR) assay

Digital PCR was performed using species-specific primers and a hydrolysis probe targeting the ITS1 region of *C*. *carpio* developed by Minamoto et al.^39^ Primer and probe specificity was evaluated in silico using BLASTn searches against sympatric cyprinids and co-occurring freshwater fishes in Thailand. No perfect matches were identified outside *C*. *carpio*, and non-target taxa contained multiple mismatches at primer 3′ ends.

*In vitro* validation used tissue DNA from common carp obtained from local markets and mucus DNA from live carp maintained at a Department of Fisheries hatchery, alongside DNA from sympatric cyprinids. Only *C*. *carpio* produced amplification signals.

Reactions were conducted on a QIAcuity Digital PCR System using 26K 24-well nanoplates. Each reaction (40 μL) contained 1× QIAcuity Probe Master Mix, 2 μL DNA template, 800 nM primers, and 400 nM probe. Cycling conditions were 95°C for 2 min followed by 45 cycles of 95°C for 15 s and 60°C for 30 s. Each sample was run in triplicate.

Positive controls (carp genomic DNA), field blanks, extraction blanks, and PCR blanks were included throughout. No amplification occurred in any negative controls. Fluorescence data were analyzed using QIAcuity Software, and absolute copy numbers were calculated from positive (k) and total (N) partitions using Poisson statistics. Raw k and *N* values are reported in Table S8.

#### Limit of detection

The limit of detection (LOD) was defined as the lowest concentration at which ≥95% of replicate reactions were positive. A 10-fold dilution series of synthetic ITS1 DNA (10^3^ copies/μL to <1 copy/μL) was tested with ten replicates per concentration. The LOD corresponded to approximately 0.15 copies per reaction. Concentrations are reported as copies per μL of DNA extract, with optional conversion to copies per liter of filtered water.

#### Occupancy modeling analysis

Bayesian hierarchical occupancy models were applied using the eDNAoccupancy RShiny application to estimate site occupancy (ψ), field detection (θ11, θ10), and laboratory detection (p11, p10) probabilities. Field replicates (three 1-L samples per site) and laboratory replicates (three dPCR reactions per sample) were modeled hierarchically. Uniform(0,1) priors were used for all parameters. Models were run with three chains of 20,000 iterations, 5,000 burn-in, and thinning of 10. Convergence was assessed using Gelman–Rubin statistics and trace plots.

Environmental covariates (temperature, pH, total dissolved solids, conductivity, turbidity) were incorporated using Bayesian variable selection. Variable importance was quantified using Posterior Inclusion Probabilities (PIPs).

### Quantification and statistical analysis

eDNA concentrations were log_10_-transformed prior to statistical analysis. Spatial and temporal effects were evaluated using two-way ANOVA with River and Year as fixed factors, followed by Tukey HSD tests. Assumptions were assessed using Shapiro–Wilk and Levene’s tests. Robustness was evaluated using aligned ranks transformation ANOVA, Kruskal–Wallis tests with Dunn–Šidák corrections, Friedman tests with Site as a block, and linear mixed-effects models with Site as a random effect. Spatial autocorrelation was assessed using global Moran’s I with 999 permutations. Statistical analyses were conducted in R, and full outputs are provided in Tables S1–S5.
