# Supplementary material for: Catching common carp with eDNA in Thailand’s rivers
Source: iScience. 2025 Dec 31;29(2):114583. doi: 10.1016/j.isci.2025.114583 (PMC12874140; doi:10.1016/j.isci.2025.114583)
Supplement: Document S1. Tables S1–S8 [file mmc1.pdf]

iScience, Volume *29*

Supplemental information

Catching common carp with eDNA in Thailand's rivers

Maslin Osathanunkul, Sarawut Ounjai, Rossarin Osathanunkul, and Panagiotis Madesis

Table S1. Two-way ANOVA results

Term	df	Sum Sq	Mean Sq	F-statistic	p-value
River	4	3.4050	0.8513	11.91	3.47×10^{-7}
Year	2	1.1352	0.5676	7.94	8.72×10^{-4}
River×Year	8	0.3473	0.0434	0.61	0.768
Residuals	60	4.2890	0.0715	-	-

Table S2. Tukey HSD post-hoc comparisons

Term	Contrast	null.value	estimate	std.error	df	statistic	adj.p.value
River	Chao Phraya - Nan	0	-0.2752	0.0976	60	-2.8185	0.0493
River	Chao Phraya - Ping	0	-0.5983	0.0976	60	-6.1283	7.37E-07
River	Chao Phraya - Wang	0	-0.1843	0.0976	60	-1.8879	0.3349
River	Chao Phraya - Yom	0	-0.4855	0.0976	60	-4.9727	5.60E-05
River	Nan - Ping	0	-0.3231	0.0976	60	-3.3098	0.0132
River	Nan - Wang	0	0.0909	0.0976	60	0.9306	0.8839
River	Nan - Yom	0	-0.2103	0.0976	60	-2.1542	0.2114
River	Ping - Wang	0	0.4140	0.0976	60	4.2404	7.24E-04
River	Ping - Yom	0	0.1128	0.0976	60	1.1555	0.7762
River	Wang - Yom	0	-0.3012	0.0976	60	-3.0849	0.0247
Year	Year2022 - Year2023	0	-0.1623	0.0756	60	-2.1459	0.0892
Year	Year2022 - Year2024	0	-0.3011	0.0756	60	-3.9810	0.0005
Year	Year2023 - Year2024	0	-0.1388	0.0756	60	-1.8352	0.1670

Table S3. Model diagnostics

Test	Statistic	df	p-value
Shapiro–Wilk	0.966	75	-
Levene (River)	1.77	4	0.144
Levene (Year)	0.48	2	0.621

Table S4. Robustness checks: non-parametric and mixed-effects models

Method	Factor	Test statistic	df	p-value	Note
**ART ANOVA**	River	F = 13.11	4, 60	9.8×10^{-8}	Significant
	Year	F = 9.85	2, 60	2.0×10^{-4}	Significant
	River×Year	F = 1.06	8, 60	0.405	ns
**Kruskal–Wallis** (per year)	River (2022)	$\chi^2 = 13.68$	4	0.0084	Significant
	River (2023)	$\chi^2 = 14.10$	4	0.007	Significant
	River (2024)	$\chi^2 = 8.16$	4	0.0859	ns
**Dunn post-hoc** (examples)	2022: Yom > Chao Phraya (p.adj = 0.017), 2023: Ping > Wang (p.adj = 0.021), Yom > Wang (p.adj = 0.005)	…	…	…	-
**Friedman**	Year	$\chi^2 = 15.58$	2	0.00041	Temporal variation significant
**Mixed-effects (lmer)**	River	$\chi^2 = 26.43$	4	2.6×10^{-5}	Site random effect not significant
	Year	$\chi^2 = 26.46$	2	2.0×10^{-6}	Significant

Table S5. Spatial autocorrelation (Moran's I)

Test	I-statistic	z-value	p-asymptotic	p-permutation
Moran's I (kNN, k=4)	0.227	2.20	0.0137	0.022

Table S6. Posterior Inclusion Probabilities (PIPs) results by covariate (temperature, pH, TDS, conductivity) across years 2022–2024.

Year	Covariate	TEM	PH	TDS	CON
2022	Ψ	0.447	0.449	0.442	0.424
2023		0.618	0.592	0.630	0.626
2024		0.608	0.599	0.650	0.664
2022	θ_{11}	0.358	0.680	0.484	0.497
2023		0.708	0.917	0.579	0.585
2024		0.582	0.502	0.619	0.591
2022	p_{11}	0.633	0.583	0.637	0.610
2023		0.607	0.440	0.501	0.373
2024		0.475	0.502	0.505	0.456
2022	θ_{10}	0.574	0.581	0.676	0.692
2023		0.625	0.621	0.727	0.757
2024		0.612	0.612	0.693	0.726
2022	p_{10}	0.825	0.577	0.426	0.412
2023		0.941	0.607	0.494	0.500
2024		0.981	0.529	0.191	0.172

Table S7. Provincial aquaculture production vs. eDNA concentrations. Province-level comparison of carp aquaculture production (FishProd 2021–2023) and eDNA concentrations (eDNA 2022–2024).

Province	eDNA 2022	FishProd 2021	eDNA 2023	FishProd 2022	eDNA 2024	FishProd 2023
Angthong	0	7403	0.965	5867	0.957	6135
Bangkok	0	6264	0	5437	0	5808
Chainat	2.868	5487	2.52	5246	3.822	5322
Chiang Mai	2.402	6133	5.512	7101	9.798	7159
Lampang	0.957	5221	1.436	3579	2.394	3459
Nakhon Sawan	1.275	15289	2.233	17582	7.020	18452
Nan	0.959	3013	2.392	3324	1.913	3591
Phayao	1.935	5835	1.274	5750	1.918	6254
Phitsanulok	2.871	8063	1.436	7300	5.744	7651
Ayutthaya	0	7821	0.955	7958	1.008	7003
Phrae	1.913	3382	2.872	3520	3.060	3854
Sukhothai	2.868	6267	3.824	6978	2.867	7103
Tak	0.478	3250	2.882	3588	0.958	3027
Uttaradit	0.956	8092	0.956	8945	0.956	7532

Table S8. Raw digital PCR (dPCR) partition counts and derived concentrations for *Cyprinus carpio* environmental DNA detections across all sites and years (2022–2024). For each sample replicate, the number of positive partitions (k) and the number of accepted partitions (N) are reported. Concentrations (copies/µL) were calculated by the QIAcuity Software Suite using Poisson statistics with ≥95% confidence intervals (CI) for each estimate.

Year	Site	cp/µL	Sample replicate	cp/µL	Partitions (Valid)	Partitions (Positive)	Partitions (Negative)
2022	1	2.867	R1	2.856	25463	3	25460
				2.914	25368	3	25365
				2.868	25480	3	25477
			R2	2.867	25352	3	25349
				2.861	25349	3	25346
				2.869	25064	3	25061
			R3	2.856	25477	3	25474
				2.853	25469	3	25466
				2.860	25370	3	25367
2022	2	1.913	R1	1.915	25473	2	25471
				1.912	23191	2	23189
				1.912	25470	2	25468
			R2	1.913	25481	2	25479
				1.911	23567	2	23565
				1.912	25487	2	25485
			R3	1.918	25455	2	25453
				1.912	25465	2	25463
				1.915	25488	2	25486
2022	3	2.915	R1	3.057	22943	3	22940
				2.882	25483	3	25480
				3.057	23895	3	23892
			R2	2.881	24586	3	24583
				2.882	25456	3	25453
				2.869	25463	3	25460
			R3	2.868	25471	3	25468
				2.872	25448	3	25445
				2.869	25484	3	25481
2022	4	1.912	R1	1.912	25474	2	25472
				1.912	25465	2	25463
				1.911	25383	2	25381
			R2	1.911	25477	2	25475
				1.913	25450	2	25448
				1.912	25352	2	25350
			R3	1.912	25462	2	25460
				1.911	25441	2	25439

				1.912	25391	2	25389
2022	5	0.956	R1	0.957	23870	1	23869
				0.955	25483	1	25482
				0.957	25412	1	25411
			R2	0.958	25475	1	25474
				0.956	25356	1	25355
				0.957	25489	1	25488
			R3	0.956	25459	1	25458
				0.955	25470	1	25469
				0.956	25434	1	25433
2022	6	0.959	R1	0.959	25467	1	25466
				0.967	25486	1	25485
				0.956	25454	1	25453
			R2	0.957	25355	1	25354
				0.961	25471	1	25470
				0.957	25403	1	25402
			R3	0.957	25447	1	25446
				0.956	25448	1	25447
				0.961	25381	1	25380
2022	7	0.956	R1	0.956	25348	1	25347
				0.955	25463	1	25462
				0.96	25368	1	25367
			R2	0.955	25338	1	25337
				0.956	25464	1	25463
				0.956	25456	1	25455
			R3	0.957	25321	1	25320
				0.956	25463	1	25462
				0.956	25481	1	25480
2022	8	0.955	R1	0.955	25454	1	25453
				0.955	25474	1	25473
				0.956	25473	1	25472
			R2	0.955	25342	1	25341
				0.955	25472	1	25471
				0.955	23822	1	23821
			R3	0.956	25490	1	25489
				0.955	25469	1	25468
				0.957	25469	1	25468
2022	9	0.959	R1	0.960	25369	1	25368
				0.959	25393	1	25392
				0.957	25430	1	25429
			R2	0.959	25461	1	25460
				0.957	25459	1	25458
				0.959	25435	1	25434
			R3	0.960	25361	1	25360

				0.959	25463	1	25462
				0.959	25368	1	25367
2022	10	0.000	R1	0	25338	0	25338
				0	25464	0	25464
				0	25456	0	25456
			R2	0	25321	0	25321
				0	25463	0	25463
				0	25481	0	25481
			R3	0	25454	0	25454
				0	25474	0	25474
				0	25473	0	25473
2022	11	1.935	R1	1.911	25488	2	25486
				1.913	25455	2	25453
				1.912	25474	2	25472
			R2	1.912	25465	2	25463
				1.912	25468	2	25466
				1.911	25488	2	25486
			R3	1.911	25490	2	25488
				1.912	25475	2	25473
				2.123	22943	2	22941
2022	12	1.913	R1	1.918	25391	2	25389
				1.911	25477	2	25475
				1.912	25472	2	25470
			R2	1.911	25488	2	25486
				1.912	25468	2	25466
				1.913	25462	2	25460
			R3	1.912	25474	2	25472
				1.918	25391	2	25389
				1.912	25465	2	25463
2022	13	2.868	R1	2.871	25448	3	25445
				2.869	25463	3	25460
				2.869	25465	3	25462
			R2	2.869	25468	3	25465
				2.869	25464	3	25461
				2.868	25471	3	25468
			R3	2.866	25490	3	25487
				2.868	25471	3	25468
				2.866	25490	3	25487
2022	14	3.830	R1	3.829	25434	4	25430
				3.830	25426	4	25422
				3.829	25431	4	25427
			R2	3.830	25430	4	25426
				3.830	25427	4	25423
				3.829	25435	4	25431

			R3	3.831	25422	4	25418
				3.831	25425	4	25421
				3.830	25429	4	25425
2022	15	2.870	R1	2.869	25463	3	25460
				2.869	25466	3	25463
				2.881	25352	3	25349
			R2	2.868	25471	3	25468
				2.866	25488	3	25485
				2.872	25434	3	25431
			R3	2.866	25486	3	25483
				2.871	25448	3	25445
				2.866	25490	3	25487
2022	16	0.961	R1	0.956	25461	1	25460
				0.957	25456	1	25455
				0.956	25463	1	25462
			R2	0.956	25477	1	25476
				0.960	25370	1	25369
				0.956	25473	1	25472
			R3	0.990	24585	1	24584
				0.959	25401	1	25400
				0.960	25372	1	25371
2022	17	0.957	R1	0.956	25473	1	25472
				0.955	25487	1	25486
				0.959	25401	1	25400
			R2	0.956	25459	1	25458
				0.957	25450	1	25449
				0.956	25470	1	25469
			R3	0.957	25434	1	25433
				0.956	25467	1	25466
				0.957	25454	1	25453
2022	18	0.956	R1	0.956	25464	1	25463
				0.955	25486	1	25485
				0.956	25477	1	25476
			R2	0.960	25360	1	25359
				0.955	25484	1	25483
				0.956	25466	1	25465
			R3	0.956	25469	1	25468
				0.957	25452	1	25451
				0.956	25474	1	25473
2022	19	1.912	R1	1.912	25474	2	25472
				1.913	25455	2	25453
				1.911	25488	2	25486
			R2	1.912	25468	2	25466
				1.911	25485	2	25483

				1.912	25464	2	25462
			R3	1.912	25469	2	25467
				1.911	25489	2	25487
				1.912	25473	2	25471
2022	20	0.956	R1	0.957	25432	1	25431
				0.956	25464	1	25463
				0.957	25451	1	25450
			R2	0.956	25468	1	25467
				0.955	25489	1	25488
				0.956	25475	1	25474
			R3	0.956	25470	1	25469
				0.957	25451	1	25450
				0.956	25472	1	25471
2022	21	0.000	R1	0	25469	0	25469
				0	25461	0	25461
				0	25450	0	25450
			R2	0	25446	0	25446
				0	25347	0	25347
				0	25465	0	25465
			R3	0	25335	0	25335
				0	25468	0	25468
				0	25448	0	25448
2022	22	2.868	R1	2.869	25463	3	25460
				2.868	25471	3	25468
				2.866	25490	3	25487
			R2	2.868	25476	3	25473
				2.866	25493	3	25490
				2.869	25465	3	25462
			R3	2.866	25486	3	25483
				2.872	25434	3	25431
				2.868	25471	3	25468
2022	23	0.000	R1	0	25490	0	25490
				0	25463	0	25463
				0	25366	0	25366
			R2	0	25336	0	25336
				0	25464	0	25464
				0	25456	0	25456
			R3	0	25321	0	25321
				0	25463	0	25463
				0	25481	0	25481
2022	24	0.000	R1	0	25453	0	25453
				0	25474	0	25474
				0	25473	0	25473
			R2	0	25342	0	25342

				0	25471	0	25471
				0	23817	0	23817
			R3	0	25490	0	25490
				0	25469	0	25469
				0	25469	0	25469
2022	25	0.000	R1	0	25369	0	25369
				0	25393	0	25393
				0	25430	0	25430
			R2	0	25461	0	25461
				0	25459	0	25459
				0	25435	0	25435
			R3	0	25471	0	25471
				0	25472	0	25472
				0	25372	0	25372
2023	1	3.825	R1	3.824	25473	4	25469
				3.824	25469	4	25465
				3.830	25431	4	25427
			R2	3.824	25469	4	25465
				3.824	25478	4	25474
				3.825	25462	4	25458
			R3	3.823	25491	4	25487
				3.824	25470	4	25466
				3.825	25461	4	25457
2023	2	2.871	R1	2.866	25487	3	25484
				2.882	25456	3	25453
				2.869	25463	3	25460
			R2	2.868	25471	3	25468
				2.872	25448	3	25445
				2.869	25484	3	25481
			R3	2.871	25448	3	25445
				2.872	25437	3	25434
				2.872	25439	3	25436
2023	3	3.830	R1	3.824	25469	4	25465
				3.825	25466	4	25462
				3.829	25442	4	25438
			R2	3.830	24431	4	24427
				3.835	25393	4	25389
				3.836	25391	4	25387
			R3	3.834	25401	4	25397
				3.830	25430	4	25426
				3.830	25429	4	25425
2023	4	11.520	R1	11.520	25359	12	25347
				11.520	25361	12	25349
				11.521	25354	12	25342

			R2	11.520	25362	12	25350
				11.520	25363	12	25351
				11.520	25359	12	25347
			R3	11.519	25366	12	25354
				11.518	25371	12	25359
				11.520	25360	12	25348
2023	5	4.781	R1	4.781	25464	5	25459
				4.782	25454	5	25449
				4.781	25466	5	25461
			R2	4.780	25469	5	25464
				4.781	25467	5	25462
				4.780	25472	5	25467
			R3	4.781	25464	5	25459
				4.780	25473	5	25468
				4.781	25465	5	25460
2023	6	1.919	R1	1.918	25384	2	25382
				1.919	25372	2	25370
				1.919	25376	2	25374
			R2	1.919	25382	2	25380
				1.920	25359	2	25357
				1.919	25373	2	25371
			R3	1.918	25402	2	25400
				1.919	25377	2	25375
				1.918	25398	2	25396
2023	7	0.957	R1	0.961	25471	1	25470
				0.955	25463	1	25462
				0.956	25448	1	25447
			R2	0.956	25459	1	25458
				0.956	25464	1	25463
				0.957	25403	1	25402
			R3	0.957	25321	1	25320
				0.957	25447	1	25446
				0.957	25355	1	25354
2023	8	1.912	R1	1.912	25475	2	25473
				1.913	25455	2	25453
				1.912	25470	2	25468
			R2	1.912	25469	2	25467
				1.912	25468	2	25466
				1.914	25452	2	25450
			R3	1.911	25488	2	25486
				1.912	25475	2	25473
				1.912	25468	2	25466
2023	9	0.956	R1	0.956	25467	1	25466
				0.956	25470	1	25469

				0.957	25459	1	25458
			R2	0.956	25471	1	25470
				0.957	25459	1	25458
				0.956	25465	1	25464
			R3	0.956	25481	1	25480
				0.957	25444	1	25443
				0.956	25367	1	25366
2023	10	0.983	R1	0.983	24772	1	24771
				0.983	24768	1	24767
				0.981	24842	1	24841
			R2	0.956	25464	1	25463
				0.956	25476	1	25475
				0.957	25439	1	25438
			R3	0.983	24775	1	24774
				0.990	24588	1	24587
				1.061	22944	1	22943
2023	11	1.274	R1	1.911	25479	2	25486
				1.911	25488	2	25486
				1.911	25488	2	25449
			R2	0.955	25483	1	25437
				0.956	25475	1	25468
				0.955	25482	1	25434
			R3	0.956	25451	1	25440
				0.957	25438	1	25453
				0.956	25469	1	25468
2023	12	2.872	R1	2.872	25435	3	25432
				2.872	25441	3	25438
				2.870	25454	3	25451
			R2	2.869	25469	3	25466
				2.869	25462	3	25459
				2.871	25447	3	25444
			R3	2.872	25490	3	25487
				2.875	25413	3	25410
				2.875	25408	3	25405
2023	13	3.824	R1	3.824	25469	4	25465
				3.825	25461	4	25457
				3.824	25472	4	25468
			R2	3.824	25476	4	25472
				3.824	25470	4	25466
				3.824	25471	4	25467
			R3	3.825	25463	4	25459
				3.825	25465	4	25461
				3.824	25474	4	25470
2023	14	2.872	R1	2.872	25439	3	25436

				2.871	25446	3	25443
				2.872	25441	3	25438
			R2	2.870	25453	3	25450
				2.870	25456	3	25453
				2.872	25434	3	25431
			R3	2.872	25439	3	25436
				2.875	25408	3	25405
				2.872	25440	3	25437
2023	15	3.828	R1	3.828	25438	4	25434
				3.829	25432	4	25428
				3.828	25441	4	25437
			R2	3.826	25457	4	25453
				3.827	25449	4	25445
				3.827	25450	4	25446
			R3	3.828	25446	4	25442
				3.828	25444	4	25440
				3.827	25451	4	25447
2023	16	0.957	R1	0.957	25456	1	25455
				0.960	25382	1	25381
				0.957	25446	1	25445
			R2	0.960	25374	1	25373
				0.957	25453	1	25452
				0.957	25442	1	25441
			R3	0.956	25463	1	25462
				0.955	25486	1	25485
				0.956	25478	1	25477
2023	17	3.826	R1	3.826	25452	4	25448
				3.825	25459	4	25455
				3.826	25455	4	25451
			R2	3.828	25441	4	25437
				3.826	25453	4	25449
				3.826	25457	4	25453
			R3	3.826	25452	4	25448
				3.827	25478	4	25474
				3.828	25443	4	25439
2023	18	0.956	R1	0.956	25472	1	25471
				0.956	25379	1	25378
				0.959	25417	1	25416
			R2	0.956	25480	1	25479
				0.955	25486	1	25485
				0.956	25462	1	25461
			R3	0.956	25474	1	25473
				0.955	25485	1	25484
				0.956	25479	1	25478

2023	19	0.000	R1	0	25474	0	25474
				0	25455	0	25455
				0	25488	0	25488
			R2	0	25468	0	25468
				0	25485	0	25485
				0	25464	0	25464
			R3	0	25469	0	25469
				0	25489	0	25489
				0	25473	0	25473
2023	20	1.912	R1	1.912	25474	2	25472
				1.912	25471	2	25469
				1.913	25458	2	25456
			R2	1.911	25479	2	25477
				1.911	25488	2	25486
				1.911	25480	2	25478
			R3	1.913	25459	2	25457
				1.912	25464	2	25462
				1.912	25467	2	25465
2023	21	0.958	R1	0.959	25416	1	25415
				0.960	25366	1	25365
				0.960	25375	1	25374
			R2	0.956	25456	1	25455
				0.956	25364	1	25363
				0.957	25465	1	25464
			R3	0.956	25335	1	25334
				0.956	25468	1	25467
				0.959	25405	1	25404
2023	22	2.546	R1	1.909	25496	2	25494
				1.910	25493	2	25491
				1.910	25489	2	25487
			R2	2.864	25486	3	25483
				2.865	25495	3	25492
				2.864	25494	3	25491
			R3	2.865	25493	3	25490
				2.864	25498	3	25495
				2.865	25493	3	25490
2023	23	0.965	R1	0.990	24586	1	24585
				0.983	24772	1	24771
				0.983	24768	1	24767
			R2	0.956	25471	1	25470
				0.955	25484	1	25483
				0.955	25487	1	25486
			R3	0.956	25462	1	25461
				0.955	25483	1	25482

				0.956	25477	1	25476
2023	24	0.955	R1	0.955	25483	1	25482
				0.956	25459	1	25458
				0.955	25484	1	25483
			R2	0.956	25462	1	25461
				0.956	25471	1	25470
				0.955	25488	1	25487
			R3	0.955	25485	1	25484
				0.955	25486	1	25485
				0.955	25483	1	25482
2023	25	0.000	R1	0	25369	0	25369
				0	25393	0	25393
				0	25430	0	25430
			R2	0	25461	0	25461
				0	25459	0	25459
				0	25435	0	25435
			R3	0	25471	0	25471
				0	25472	0	25472
				0	25372	0	25372
2024	1	9.561	R1	9.560	25472	10	25462
				9.561	25467	10	25457
				9.561	25470	10	25460
			R2	9.561	25469	10	25459
				9.560	25472	10	25462
				9.560	25475	10	25465
			R3	9.561	25468	10	25458
				9.561	25466	10	25456
				9.561	25469	10	25459
2024	2	4.781	R1	4.781	25464	5	25459
				4.782	25457	5	25452
				4.782	25460	5	25455
			R2	4.781	25466	5	25461
				4.782	25459	5	25454
				4.781	25465	5	25460
			R3	4.780	25474	5	25469
				4.781	25466	5	25461
				4.781	25468	5	25463
2024	3	9.558	R1	9.558	25479	10	25469
				9.558	25481	10	25471
				9.559	25476	10	25466
			R2	9.558	25482	10	25472
				9.558	25478	10	25468
				9.558	25479	10	25469
			R3	9.560	25474	10	25464

				9.558	25480	10	25470
				9.558	25481	10	25471
2024	4	15.29	R1	15.29	25469	16	25453
				15.30	25461	16	25445
				15.29	25475	16	25459
			R2	15.30	25466	16	25450
				15.29	25471	16	25455
				15.29	25474	16	25458
			R3	15.31	25460	16	25444
				15.29	25472	16	25456
				15.29	25471	16	25455
2024	5	0.959	R1	0.957	5442	1	5441
				0.957	25438	1	25437
				0.959	25385	1	25384
			R2	0.960	25361	1	25360
				0.961	25352	1	25351
				0.960	25377	1	25376
			R3	0.959	25401	1	25400
				0.959	25413	1	25412
				0.958	25382	1	25381
2024	6	2.869	R1	2.868	25471	2	25469
				2.869	25463	2	25461
				2.869	25465	2	25463
			R2	2.875	25408	2	25406
				2.872	25438	2	25436
				2.872	25441	2	25439
			R3	2.866	25493	2	25491
				2.867	25482	2	25480
				2.866	25491	2	25489
2024	7	0.957	R1	0.957	25454	1	25453
				0.957	25447	1	25446
				0.956	25448	1	25447
			R2	0.956	25463	1	25462
				0.956	25477	1	25476
				0.957	25443	1	25442
			R3	0.957	25452	1	25451
				0.957	25439	1	25438
				0.957	25456	1	25455
2024	8	2.881	R1	2.880	25367	3	25364
				2.881	25354	3	25351
				2.880	25369	3	25366
			R2	2.882	25344	3	25341
				2.881	25360	3	25357
				2.881	25358	3	25355

			R3	2.882	25347	3	25344
				2.882	25351	3	25348
				2.881	25361	3	25358
2024	9	2.869	R1	2.870	25457	3	25454
				2.870	25452	3	25449
				2.871	25443	3	25440
			R2	2.869	25464	3	25461
				2.869	25467	3	25464
				2.868	25472	3	25469
			R3	2.866	25486	3	25483
				2.867	25481	3	25478
				2.867	25484	3	25481
2024	10	0.957	R1	0.959	25385	1	25384
				0.957	25438	1	25437
				0.957	25452	1	25451
			R2	0.956	25464	1	25463
				0.956	25476	1	25475
				0.957	25439	1	25438
			R3	0.957	25450	1	25449
				0.955	25482	1	25481
				0.957	25446	1	25445
2024	11	1.918	R1	1.919	25381	2	25379
				1.919	25372	2	25370
				1.920	25366	2	25364
			R2	1.918	25395	1	25394
				1.919	25379	1	25378
				1.919	25382	1	25348
			R3	1.917	25408	1	25354
				1.918	25389	1	25347
				1.917	25411	1	22691
2024	12	3.013	R1	2.882	25349	3	25346
				2.881	25355	3	25352
				2.882	25348	3	25345
			R2	3.071	22692	3	22689
				3.067	23793	3	23790
				3.104	22005	3	22002
			R3	3.054	23899	3	23896
				3.082	22341	3	22338
				3.091	22064	3	22061
2024	13	2.867	R1	2.867	25479	3	25476
				2.867	25482	3	25479
				2.868	25475	3	25472
			R2	2.866	25488	3	25485
				2.866	25490	3	25487

				2.867	25483	3	25480
			R3	2.868	25477	3	25474
				2.869	25463	3	25460
				2.868	25471	3	25468
2024	14	6.708	R1	6.692	25474	7	25467
				6.692	25471	7	25464
				6.693	25467	7	25460
			R2	6.835	23943	7	23936
				6.692	25477	7	25470
				6.692	25474	7	25467
			R3	6.692	25472	7	25465
				6.693	25467	7	25460
				6.692	25475	7	25468
2024	15	3.823	R1	3.822	25483	4	25479
				3.823	25475	4	25471
				3.823	25480	4	25476
			R2	3.823	25475	4	25471
				3.825	25459	4	25455
				3.825	25462	4	25458
			R3	3.823	25479	4	25475
				3.824	25469	4	25465
				3.823	25481	4	25477
2024	16	0.960	R1	0.960	25374	1	25373
				0.960	25382	1	25381
				0.961	25353	1	25352
			R2	0.960	25374	1	25373
				0.961	25351	1	25350
				0.960	25372	1	25371
			R3	0.960	25382	1	25381
				0.959	25407	1	25406
				0.959	25396	1	25395
2024	17	2.866	R1	2.867	25479	3	25476
				2.868	25477	3	25474
				2.867	25485	3	25482
			R2	2.866	25486	3	25483
				2.867	25479	3	25476
				2.866	25491	3	25488
			R3	2.866	25493	3	25490
				2.866	25487	3	25484
				2.865	25493	3	25490
2024	18	0.956	R1	0.957	25438	1	25437
				0.956	25475	1	25474
				0.956	25463	1	25462
			R2	0.956	25471	1	25470

				0.956	25466	1	25441
				0.957	25442	1	25465
			R3	0.956	25463	1	25462
				0.956	25477	1	25476
				0.955	25484	1	25483
2024	19	4.780	R1	4.780	25471	5	25466
				4.781	25465	5	25460
				4.781	25468	5	25463
			R2	4.779	25479	5	25474
				4.779	25482	5	25477
				4.780	25472	5	25467
			R3	4.781	25467	5	25462
				4.780	25473	5	25468
				4.780	25469	5	25464
2024	20	16.282	R1	16.28	25442	17	25425
				16.28	25434	17	25417
				16.29	25420	17	25403
			R2	16.28	25438	17	25421
				16.27	25446	17	25429
				16.28	25441	17	25424
			R3	16.28	25434	17	25417
				16.29	25433	17	25416
				16.29	25429	17	25412
2024	21	0.956	R1	0.956	25463	1	25462
				0.956	25471	1	25470
				0.958	25426	1	25425
			R2	0.957	25439	1	25438
				0.956	25368	1	25367
				0.957	25451	1	25450
			R3	0.955	25485	1	25484
				0.956	25460	1	25459
				0.956	25475	1	25474
2024	22	3.822	R1	3.822	25481	4	25477
				3.823	25477	4	25473
				3.823	25479	4	25475
			R2	3.823	25474	4	25470
				3.822	25482	4	25478
				3.823	25480	4	25476
			R3	3.822	25484	4	25480
				3.822	25481	4	25477
				3.822	25483	4	25479
2024	23	0.957	R1	0.957	24438	1	24437
				0.957	24451	1	24450
				0.957	24447	1	24446

			R2	0.956	25471	1	25470
				0.958	25424	1	25423
				0.958	25432	1	25431
			R3	0.956	25463	1	25462
				0.957	25452	1	25451
				0.957	25439	1	25438
2024	24	1.008	R1	0.991	24412	1	24411
				0.990	24586	1	24585
				0.990	24589	1	24588
			R2	0.989	24612	1	24611
				0.991	25415	1	25414
				0.990	24592	1	24591
			R3	1.061	22943	1	22942
				0.990	24583	1	24582
				1.077	22014	1	22013
2024	25	0.000	R1	0	25369	0	25369
				0	25393	0	25393
				0	25430	0	25430
			R2	0	25461	0	25461
				0	25459	0	25459
				0	25435	0	25435
			R3	0	25471	0	25471
				0	25472	0	25472
				0	25372	0	25372
